# Complete Removable Denture Retained by a Symphyseal Single Implant: A Case Report

**DOI:** 10.7759/cureus.54938

**Published:** 2024-02-26

**Authors:** Akkar Youness, Nadia Merzouk, Anissa Regragui

**Affiliations:** 1 Department of Dentistry, Faculty of Dental Medicine, Mohammed V University, Rabat, MAR; 2 Department of Prosthodontics, Faculty of Dentistry, Mohammed V University, Rabat, MAR

**Keywords:** mandibular complete denture, symphyseal single implant, single implant, mandibular overdenture, complete denture

## Abstract

Complete denture is a real challenge for any practitioner. The aim of implantology is to optimize prosthetic balance and ensure that prostheses are perfectly integrated from a bio-functional viewpoint, despite an unfavorable anatomophysiological context. Here, we present a case managed in our department concerning a mandibular complete removable denture retained by a medial symphyseal implant.

A 61-year-old fully edentulous mandibular patient with a Kennedy Applegate class I edentulous maxilla in good general health consulted for the renewal of his mandibular complete denture, which was deemed unstable and non-retentive. The exo-oral examination was normal. The mandibular crest was heavily resorbed, especially posteriorly, and covered with slightly inflammatory fibromucosa. The patient refused bone augmentation surgery. We performed a piezographic mandibular prosthesis retained by a single standard implant at the level of the mandibular symphysis. The prosthesis was stable and retentive. Masticatory comfort and efficiency were satisfactory and the psychological integration of the prosthesis was improved.

Several studies have shown that a single symphyseal implant is a therapeutic alternative that completes the therapeutic range in specific cases. It should be limited to the mandibular arch in elderly patients with reduced bone volume. Given the lack of randomized controlled trials, routine use of this new approach is not recommended, and further studies are required.

## Introduction

The success of prosthetic rehabilitation is based on respect for the triad of equilibrium, which consists of stabilization, support, and retention, thus ensuring the durability of the prosthesis and its integration on a psychological and physiological level [1]. However, this balance is compromised in cases of significant resorption and a reduction in the extent of the bearing surface, with or without significant tonicity of the lingual and perioral muscles; problems are often observed in the mandible [2]. For this reason, the use of complementary retention devices, namely, implants, when conditions allow is a valuable means of optimizing this balance. However, implant-supported complete removable prostheses depend on the pre-prosthetic design of the bone site and optimal preparation of the peri-implant tissues. Elderly patients are often reluctant to undergo bone reconstruction surgery because of its complexity and the additional cost involved. In addition, maintaining satisfactory oral hygiene often requires greater manual dexterity and visual acuity. A removable prosthesis stabilized on two implants in canine or premolar sites is a minimum requirement according to the 2002 McGill consensus [3,4].

However, in certain situations of atrophied mandibular ridges that do not offer the possibility of implantation at the canine or premolar sites, a recently developed therapeutic approach proposes the use of a single implant in the medial symphyseal region as a means of retention complementary to the mandibular complete denture [2]. Here, we report a case of oral rehabilitation using a mandibular complete denture retained by a single medial symphyseal implant, as well as a review of the literature on this subject.

## Case presentation

**Figure 1:**
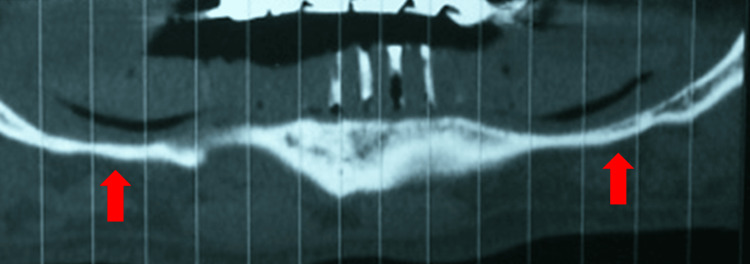
DentaScan with X-ray guide revealing a thin posterior ridge.

A 61-year-old complete edentulous unimaxillary mandibular patient with a Kennedy Applegate Class I edentulous maxilla wearing a composite prosthesis with an anterior bridge from 13 to 23 in good general health consulted for the renewal of his mandibular complete denture, which was judged to be unstable and non-retentive. The exo-oral examination was normal. The mandibular crest was heavily resorbed, especially posteriorly, and covered with slightly inflammatory fibromucosa. The small prosthetic bases did not use the entire bearing surface. The DentaScan (Figure 1) showed very pronounced resorption of the mandibular crest, especially in the posterior sectors, proximity of the right and left inferior alveolar nerves, and very low mandibular bone height except in the medial symphyseal region.

Given the patient’s financial means, age, anatomophysiological conditions, and refusal to undergo major bone augmentation surgery, the solution chosen was to perform a complete denture using a single medial symphyseal implant.

Surgical steps

After disinfection of the mucocutaneous field, local anesthesia was applied to the affected site. As the ridge was narrow, a small flap was lifted (crestal incision) to expose the underlying bone, which was then prepared using a ball cutter before the pilot drill could be used.

**Figure 2:**
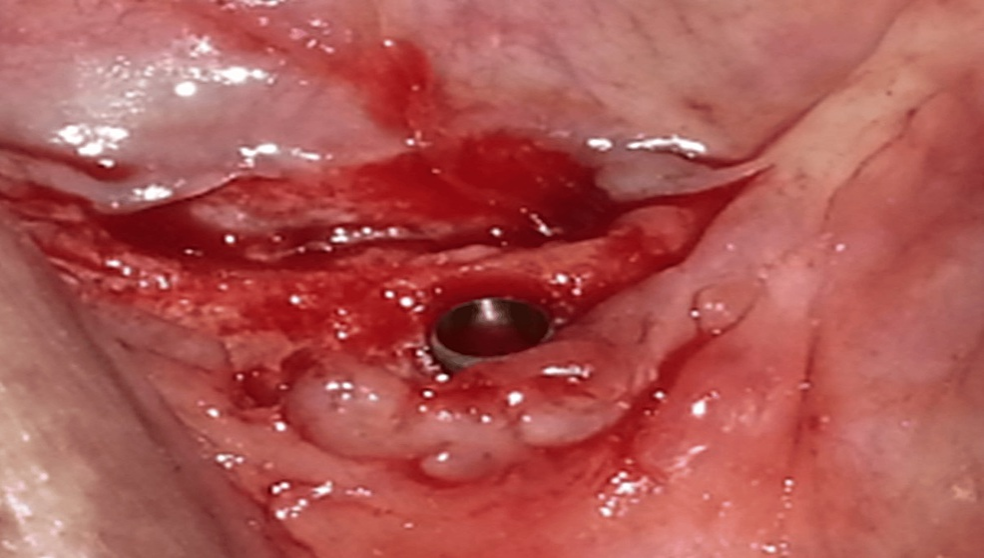
Fully inserted implant.

The drilling of the implant compartment, dictated by the surgical guide, was performed under external irrigation with the pilot drill (Biotech System) mounted on a contra-angle. The implant was then inserted into the implant compartment (Figure 2). The flap was repositioned and sutured with a silk thread to obtain a perfect coaptation of the banks. The first time being placed in a nurse was three months, and then a healing screw was put in place during the second surgery. Good osseointegration was achieved. To avoid transmitting harmful forces to the implant, the prosthesis was hollowed out in front of the healing screw and rebased with a transitional material such as Kerr’s Fitt for six weeks.

Prosthetic steps

**Figure 3:**
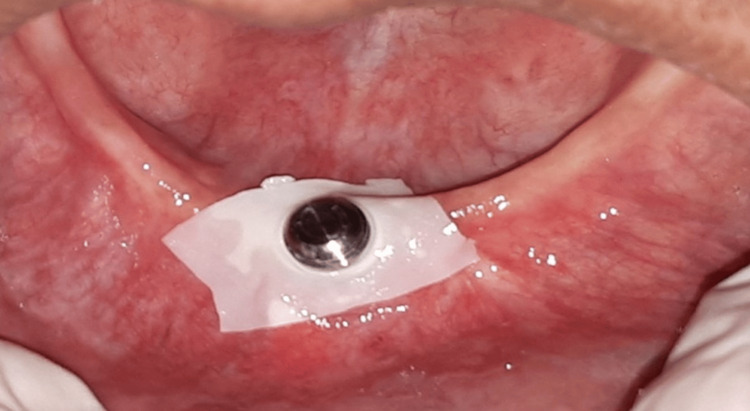
Solidarization in the mouth of the counterpart of the attachment.

**Figure 4:**
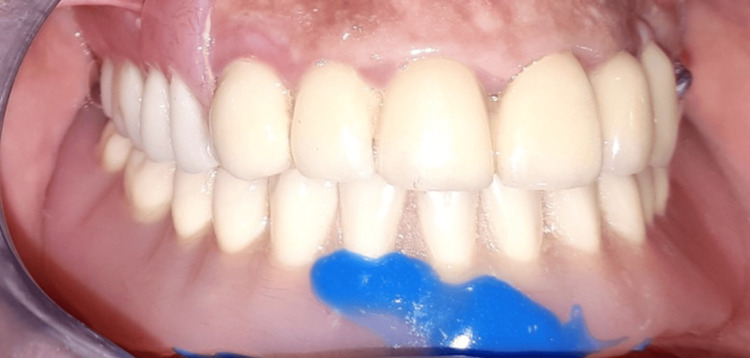
The final result.

Then, the healing screw was replaced by a 6 mm high Locator® abutment. The solidarization of the counterpart of the attachment was done directly in the mouth (Figure 3). The control of the occlusion during the mouthing and follow-up as well as balancing steps were conventional (Figure 4). Appropriate hygiene advice was given. The mandibular complete denture retained by a medial symphyseal implant was stable and retentive. Comfort and masticatory efficiency were regained, along with better psychological integration of the prosthesis. The bone level around the implant was stable four years after the placement of the device, with no complaints from the patient.

## Discussion

Completely edentulous patients frequently suffer from instability of their mandibular prostheses and lack of retention. Most of these patients have a preference for the supra-implant prosthesis as an economical, aesthetically acceptable, and easily applicable therapeutic solution [5,6]. Many studies have reported significant medium- and long-term success rates [4-7]. It should be noted that opinions differ regarding the appropriate number of implants to support a mandibular complete denture [2-7]. Currently, it is accepted that in cases of significant resorption of the mandibular crest, the supra-radicular complete removable prosthesis, stabilized by at least two symphyseal implants connected by a conjunction bar or axial attachment supports, is the reference treatment [3)]. In our case, we performed a mandibular complete denture retained by a single medial symphyseal implant. The results after three months of follow-up showed that it is possible to use a single medial symphyseal implant connected to an axial attachment supporting the mandibular complete denture in specific cases (elderly patient, resorbed crest, refusal of bone graft surgery, etc.). Compared with the conventional complete denture previously performed in this patient, the increased retention and stabilization provided by the implant significantly improved patient comfort and masticatory efficiency. The chin symphysis is a good implant site in terms of bone quality and volume [4-8]. The symphyseal region is easily accessible and implant placement requires minimal working time and generates little trauma.

Maintenance is easy. The major disadvantage is the development of sagittal, transverse, and vertical axes of rotation. This problem can be solved by creating a prosthesis that respects the prosthetic corridor while increasing the lateral (sublingual) extensions as much as possible [9]. Implant failures are rare [10]. Several parameters must be taken into account for this treatment to be successful [11], including good bone density (bone type I or II); sufficient bone height and width >6/12 mm; an implant with an improved surface finish, height ≥8 mm, diameter ≥3.75 mm; a two-staged surgical protocol; respect for the healing phase; choice of attachment (high and wide depending on the prosthetic space available); the design and construction of the complete denture according to the imperatives of balance; antagonistic arch support for complete denture; and regular prosthetic and implant maintenance. The long-term success rate and the degree of bone loss around the implant are comparable to those observed when the mandibular complete denture is retained by two symphyseal implants [10].

## Conclusions

The implant-supported complete removable prosthesis is an additional tool in the therapeutic arsenal of total edentulism. It makes it possible to manage cases for which there were no satisfactory solutions offered by conventional complete dentures due to atrophied mandibular ridges and the advanced age of the patient limiting any overly invasive surgery. It should be noted that there are no randomized controlled clinical trials, which does not allow us to recommend the routine use of this new approach. Further studies are needed. The complete mandibular removable prosthesis retained by a medial symphyseal implant should not replace the one retained by two implants at the symphyseal level on either side of the medial sagittal axis but complete the therapeutic range in certain specific cases. It is necessary to limit ourselves to the mandibular arch in patients who do not have excessive masticatory forces or parafunctions.

## Figures and Tables

**Figure 5:**
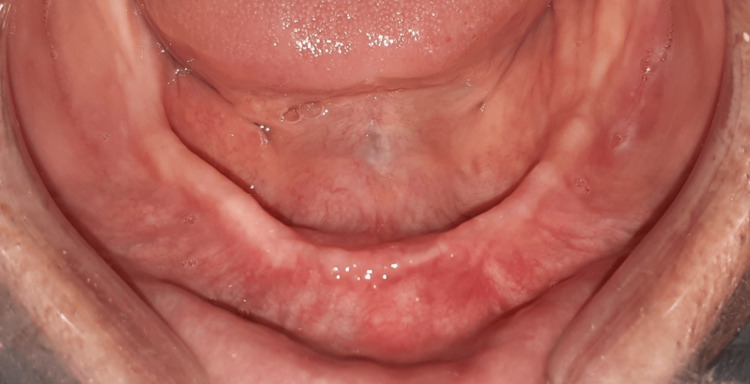
Preoperative endobuccal image showing a posterior resorbed mandibular crest.

**Figure 6:**
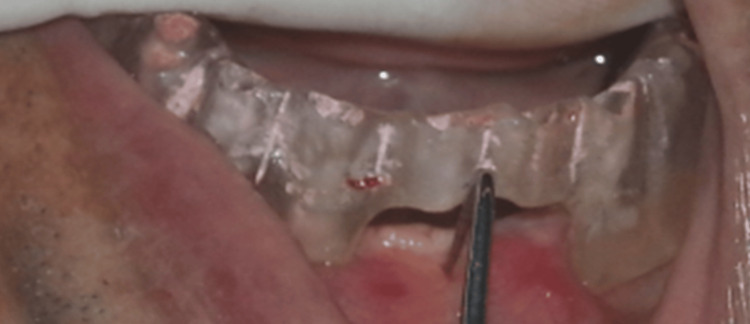
The use of a surgical guide before implantation.

**Figure 7:**
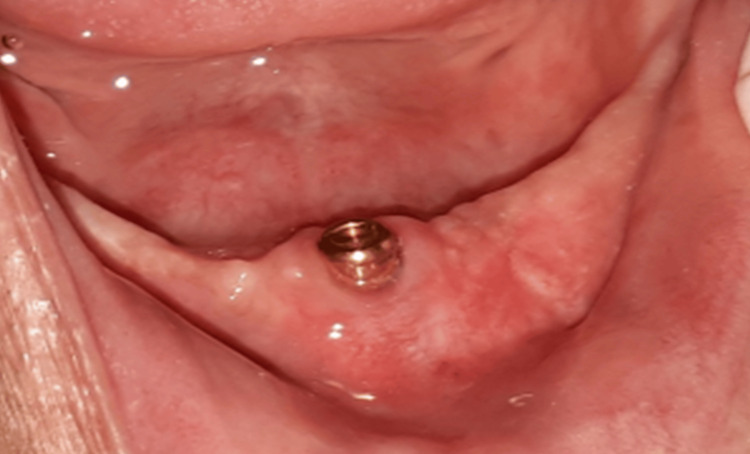
Fitting the healing screw.

**Figure 8:**
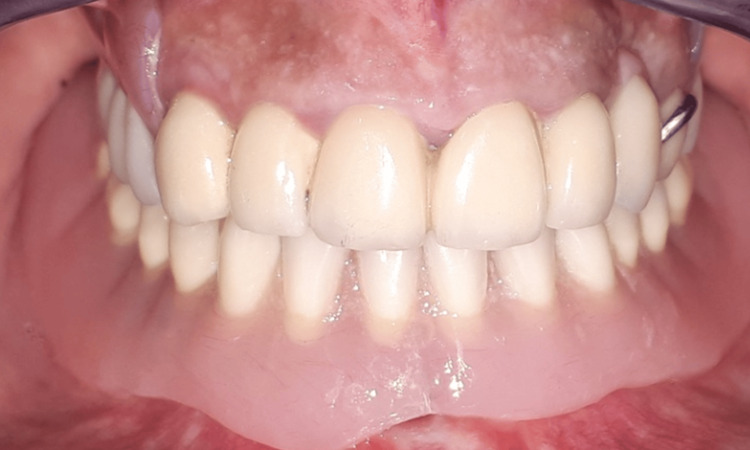
Insertion of the prosthesis and control of the occlusion.
